# Development of nursing handoff competency scale: a methodological study

**DOI:** 10.1186/s12912-024-01925-w

**Published:** 2024-04-24

**Authors:** Jiyoung Do, Sujin Shin

**Affiliations:** 1 College of Nursing, Catholic University of Pusan, 74 Oryundae-ro, Geumjeong-gu, 46265 Busan, Korea; 2 College of Nursing, Ewha Womans University, 52 Ewhayeodae-gil, Seodaemun-gu, 03760 Seoul, Korea

**Keywords:** Nursing, Education, Nursing handoff, Instrument validation, Competency

## Abstract

**Background:**

Nursing handoff competency is the ability of the nurse performing the handoff to select and interpret the necessary information for patient care and to convey it efficiently to the nurse accepting the handoff. Nursing handoff is an important nursing task that ensures nursing care continuity, quality and patient safety. This study aimed to develop a scale to measure nursing handoff competency and verify its validity and reliability.

**Methods:**

This study adopted a methodological design. A research process included three phases: (1) scale development (literature review and interviews); (2) scale validation (validity and reliability); (3) standard setting. Data were collected from 496 clinical nurses currently working in hospital wards, intensive care units, and emergency rooms, and who independently perform a handoff in South Korea.

**Results:**

The final scale comprises a self-reported 4-points Ilert scale with 25 items based on four factors: knowledge on handoff methods, identification of patient information, judgment and transfer of nursing situation, and “formation of supportive relationships. Construct validity, criterion-related validity, and discrimination validities were verified and the fitness of the scale revealed good results in confirmatory factor analysis. The Cronbach’s α of the whole tool was.912 and the cut-off score for satisfied/unsatisfied was.72.

**Conclusions:**

The developed scale can evaluate the nurse’s handoff competencies and determine whether training is necessary. The measurement results of the scale can be used to select training subjects and compose the contents of the education program.

**Supplementary Information:**

The online version contains supplementary material available at 10.1186/s12912-024-01925-w.

## Introduction

Nursing Handoff is communication between nurses sharing patient information [1]. The Joint Commission on Accreditation of Healthcare Organizations defines a handoff as an interactive process of transferring patient-specific information to ensure patient care continuity and patient safety [2]. The nursing care of inpatients is transferred between nurses at least 2–3 times every 24 h, but nurses must consistently provide proper care [3]. Therefore, nursing handoff is one of the most important nursing tasks in ensuring patient safety and quality of care.

The nurse who is accepting the handoff (receiving nurse) understands the patient’s situation based on the content and delivery method of the information provided by the nurse performing the handoff (sending nurse) [4]. In particular, the information content and delivery method affect the receiving nurse’s clinical judgment, which is directly linked to patient care. Hence, the nurse must identify significant information and effectively deliver it [5]. Competencies required for this can be divided into two categories: the ability to comprehensively understand the patient’s health problems by analyzing patient information and the ability to explain things so that nurses can easily understand them.

Previous studies used clinical reasoning competency [6], critical thinking disposition [7], clinical judgment [4], communication ability [8], and communication clarity [9] to indirectly measure the handoff competency. These tools do not reflect the characteristics of the nursing handoff, such as accurately grasping important patient information, presenting it logically, and delivering it promptly and accurately. Therefore, these instruments have limitations in accurately assessing handoff competency.

The Handoff Clinical Evaluation Exercise [10] and Handover Evaluation Scale [11] were developed to not contain items to assess the specific abilities required for a handoff. The Korean versions of the Perceived self-efficacy of hand-off reporting scale [12] and scale to evaluate communication in nursing handoff [13] reflect the handoff characteristics but only focus on specific areas such as communication standards. Therefore, an instrument that addresses the limitations of existing scales and comprehensively assesses nursing handoff competency is needed.

This study aimed to develop a nursing handoff competency scale for assessing the overall handoff competencies required of nurses to identify meaningful patient information and deliver it effectively. The assessment results can be used to identify nurses who need additional training and shed light on their areas of weakness to develop education programs specific to their needs. Providing tailored educational support can enhance the nursing handoff competencies, which would ultimately improve nursing care efficiency and quality.

## Methods

### Study design

This methodological study developed a scale to measure nursing handoff competency and to verify its validity and reliability.

### Study procedure

This study was conducted in three phases: developing nursing handoff competency measurement scale, testing its validity and reliability, and standard setting for the scale.

#### Phase 1: Development of the scale

The conceptual framework was derived through literature and interviews. From this, preliminary items were formed.

##### Conceptual framework

Anayzing the literature review and interview results generated the conceptual framework. We searched using the following search criteria. Literature published from 2011 to 2021 was searched and searches were performed on PubMed, CINAHL, RISS, KISS. The terms used in the search were a combination of handoff, handover, nursing, competency, and competence. A literature review was conducted using 26 studies on intervention studies evaluating the effectiveness of handoff education programs, systematic literature review studies, and studies on the development of patient handoff scales.

Personal interviews were conducted with 2 nursing managers and 10 clinical nurses to derive qualitative data. To confirm suitability with the clinical department, interviews were conducted with a total of 12 people, including nurse managers, new nurses, and clinical nurses with preceptor experience. To specifically analyze handoff capabilities, intensive care unit nurses who intensively care for patients with complex health problems were included, and ward and emergency room nurses were also included. The main questions of the interview were open-ended: “What does it mean to be good at handoff?” and “What are the characteristics of a nurse who is good at handoff?” The inductive content analysis method of Elo and Kyngäs [14] was used for data analysis. The analysis classified the conceptual framework of nursing handoff competency into two dimensions: nursing judgment and nursing delivery.

##### Content validation of the preliminary items

Content validity was conducted in two rounds for 2 dimensions, 8 attributes, and 69 preliminary items derived from the conceptual framework phase. The first round was conducted by 9 experts, including nursing college professors, nursing managers, and nurses with > 5 years of nurse education experience. The evaluation results were analyzed whether the Item-Content Validity Index (I-CVI) of the item was > 0.80 and the S-CVI/Ave was > 0.90 [15]. S-CVI/Ave was 0.92 in the first round, which satisfied the validity standard. The I-CVI distribution was 0.67 ∼ 1.00 and with 10 items having < 0.80.

Six items that overlapped with other items or were inappropriate for measuring handoff competence were deleted among the items with an I-CVI of < 0.80. Additionally, four items with unclear meanings were modified. The second content validity was qualitatively evaluated by three nursing professors. Items with similar contents were deleted, and items were organized to include only the core contents. Finally, 2 dimensions, 8 attributes, and 49 preliminary questions were derived.

#### Phase 2: Evaluation of the scale

In the scale verification stage, construct validity, convergent validity, discriminant validity, criterion validity, and reliability were verified.

##### Samples and data collection

Data collection was performed twice for tool evaluation. The first survey was conducted for exploratory factor analysis (EFA) in the scale development stage, and the second survey was conducted for confirmatory factor analysis (CFA), convergent, discriminant, criterion validities, and reliability verification in the scale verification stage.

The inclusion criteria for this study are as follows: (1) nurses currently working in hospital wards, intensive care units, and emergency rooms, and (2) clinical nurses performing handoff independently. The exclusion criteria of this study are as follows: (1) nurses currently on duty at the hospital but not performing patient handoff, and (2) new nurses who do not perform independent nursing due to the training period. Participants in the first main survey were excluded from the second main survey.

Based on previous studies, the number of samples for EFA is required to be 5–10 times greater than the number of items in the scale [16], which was calculated as 273, considering the dropout rate of 10%. The number of samples for CFA was calculated to be 223, considering the appropriate research results of at least 200 people and a dropout rate of 10%. The second main survey was conducted with the scales modified as an EFA result with the data collected in the first main survey.

For data collection, the researcher explained the purpose of the study and the personal information protection of research participants on the social network system and online cafes, where clinical nurses are the main visitors, and posted the link address of the recruitment documents and questionnaires for research participants. The first survey included 273 participants, while the second included 223, with a total of 496 participants.

##### Measurement

The general characteristics of the participants, including gender, age, type and region of the institution, department, total clinical career, clinical career at the current hospital, and current working type were investigated. The first survey consisted of 57 questions, including 49 preliminary items and 8 general characteristic items. The second survey consisted of 65 items, including 27 items modified after EFA, 30 for criterion validity, and 8 for general characteristics.

The Korean version of the “Nurses Clinical Reasoning Scale (NCRS)” [17] and the “Global Interpersonal Communication Competence” measuring communication ability (GICC-15) scale were used to verify the criterion validity [18]. NCRS consists of 15 items on a 5-point Likert scale, wherein higher scores indicate higher clinical reasoning competencies. The scale’s reliability was Cronbach’s α = 0.94; in this study, Cronbach’s α was 0.87. The GICC-15 consists of 15 items on a 5-point Likert scale, wherein higher scores indicate higher communication abilities. The scale’s reliability was Cronbach’s α = 0.72; in this study, Cronbach’s α was 0.77.

##### Data analysis

The collected data were analyzed using SPSS 25.0 and AMOS 22.0 programs. The specific analysis method is as follows.

1. The general participant characteristics were analyzed using descriptive statistics such as frequency, percentage, mean, and standard deviation.
2. EFA and CFA were conducted to verify construct validity. Kaiser-Meyer-Olkin (KMO) measure and Bartlett’s test were conducted to confirm that the items were suitable for EFA. The maximum likelihood method was used for factor extraction, and the promax rotation method was used for factor rotation. The criteria for deleting items were a factor loading value of less than 0.30 [19]. Model fit in CFA was judged through the ${x}^{2}$test (*p* >.05), Standardized Root Mean-square Residual (SRMR < 0.05), Root Mean Square Error of Approximation (RMSEA < 0.05), Comparative Fit Index (CFI > 0.9), and Tucker-Lewis Index (TLI > 0.9).
3. The standardized factor loading values derived from CFA and the correlation coefficient between factors and standard error values were used to confirm the confidence interval of the correlation coefficient between factors to verify convergent and discriminant validities.
4. Pearson’s correlation coefficient was used to verify the criterion validity.
5. the reliability of the developed scale was evaluated using Cronbach’s α value and Spearman-Brown’s coefficient.

### Phase 3: Standard setting of the scale

Finally, the cut-off score of the developed scale was set. The extended Angoff method estimates the score expected for a minimum competency holder [20]. The criteria for sufficiency/insufficiency in this study were established using the extended Angoff method. Criteria setters consisted of 6 people, including 2 new nurses and 4 experienced nurses who had experience in educating new nurses. The first round score was calculated by assigning a score that a nurse with a minimum competency could obtain out of four points for each item on the scale. A discussion was conducted between the criteria setters in the second round, based on the evaluation results of the first round. The cut-off score was derived through a total of two rounds.

### Ethical consideration

#### Ethical approval

for this study was obtained from the institutional review board of Ewha Womans University (Approval no. ewha-202105-0028-02). The researcher provided sufficient information to the participants about the purpose of the study, the use of data, and the protection of information before the survey. Consent was obtained from all participants in the online survey by presenting a consent form and having them participate in the survey after agreeing to the study.

## Results

### Development of the initial items

Based on the literature, a concept analysis resulted in the derivation of two dimensions: nursing judgment and nursing delivery, as components for assessing nursing handoff competencies. In previous studies, handoff competencies of nursing judgment dimension include clinical reasoning, problem-solving ability, critical thinking tendency, clinical judgment, handoff performance ability, clinical performance ability, handoff self-efficacy, communication self-efficacy, handoff confidence, reporting confidence. Nursing delivery dimensions were evaluated as communication clarity, communication ability, information clarity, SBAR skills and knowledge, and handoff time. Notable indicators from scales utilized in prior research to measure nursing handoff capabilities were analyzed. Additionally, characteristics and items were derived through studies on factors influencing handoff, studies on the development of handoff scale, and qualitative studies on nurses’ experiences with handoff. Thus, in the nursing judgment dimension, 5 attributes and 17 indicators were generated, while in the nursing delivery dimension, 3 attributes and 17 indicators were derived.

Furthermore, through the analysis of interview content, two dimensions, nursing judgment, and nursing delivery, were developed, yielding 8 attributes and 48 indicators. Within the nursing judgment dimension, 5 attributes and 23 indicators were derived. These attributes encompass possessing substantial knowledge related to patient conditions, understanding and applying hospital-suggested handoff methods, and comprehending meaningful information. Moreover, an attribute not previously explored in the literature, “utilizing various resources for information gathering,” was identified. The attribute “reasoning health problems by considering contextual situations” includes indicators for task prioritization, comprehensive understanding of patient health issues, and identification of contextual factors influencing patient condition changes.

In the developmental phase of the conceptual framework, attributes and indicators derived from literature and interviews were analyzed, similar content was grouped, and duplicated indicators were eliminated. Consequently, the initial scale comprised two dimensions, eight attributes, and 69 preliminary items.

### Participant characteristics

The general characteristics of the participants in this study are shown in Table 1. The first survey included 228 (83.5%) female participants and an average age of 29.7 (± 5.0) years. The tertiary general hospital was the most common type of hospital where the participants worked 148 (54.2%), and the average clinical career was 4.8 (± 3.9) years. Additionally, 85.7% of the participants in the second main survey were female, the average age was 29.9 (± 4.8) years, and the average clinical career was 4.4 (± 3.5) years.

**Table 1:** General characteristics of participants (*N* = 496)

Characteristics		First survey (*n* = 273) n(%) or M ± SD			Second survey (*n* = 223) n(%) or M ± SD	
Gender	Female		228	(83.5)	191	(85.7)
	Male		45	(16.5)	32	(14.3)
Age (years)			29.7 ± 5.0		29.9 ± 4.8	
Clinical career (years)			4.8 ± 3.9		4.4 ± 3.5	
Type of hospital	Tertiary general hospital		148	(54.2)	81	(36.3)
	General hospital		91	(33.3)	74	(33.2)
	Hospital		28	(10.3)	63	(28.3)
	Long term care hospital		6	(2.2)	5	(2.2)

### Validity and reliability

#### Construct validity

##### Exploratory factor analysis

**Fig. 1:**
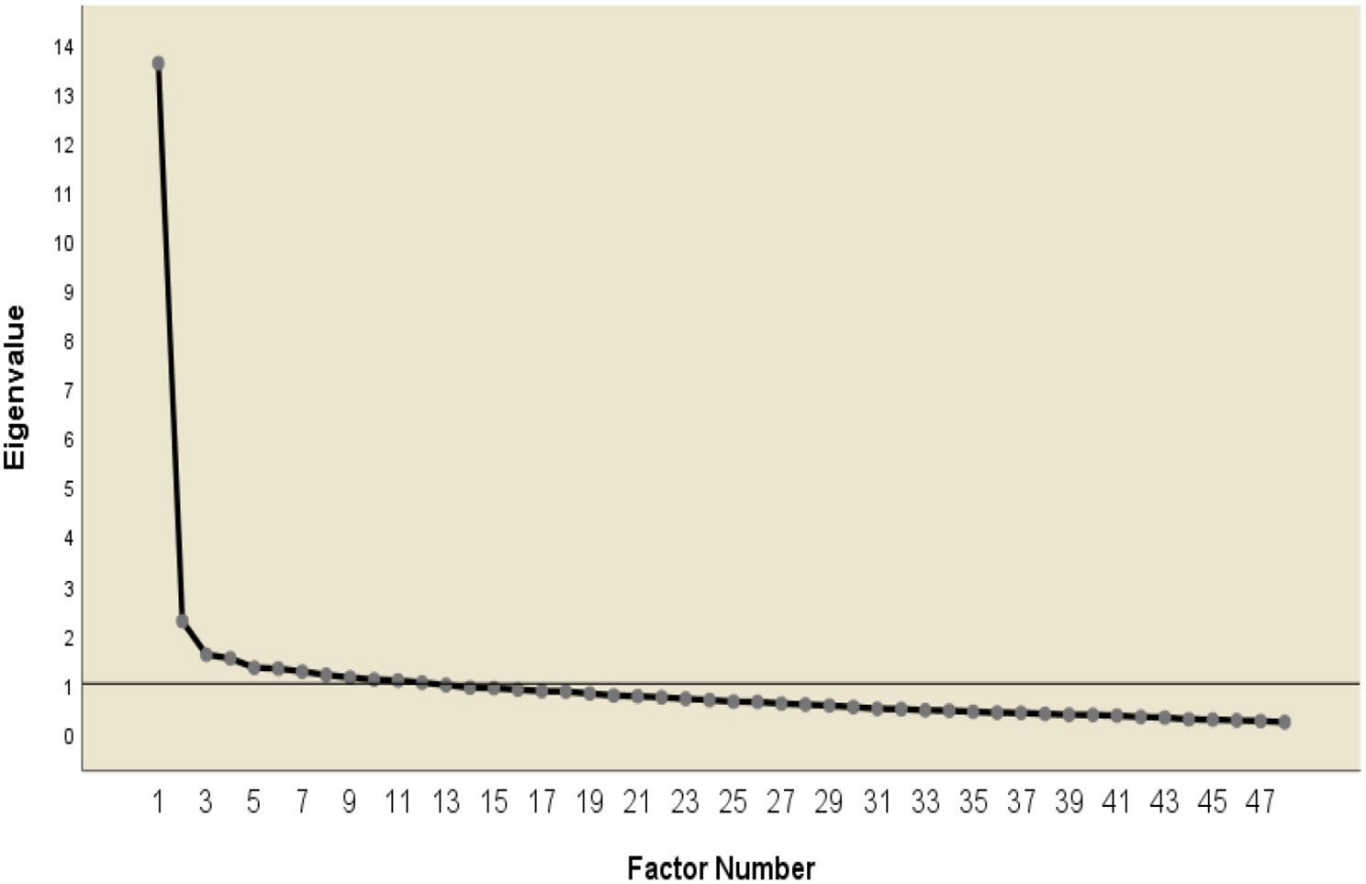
Scree plot eigenvalues of exploratory factor analysis

The correlation coefficient for the item “I do not talk about things that are not related to work” between the item and total score was *r* =.14. The reliability of all items increased from Cronbach’s α of 0.943 to 0.944 when this item was deleted. Therefore, EFA was performed on 48 questions except for this one. The KMO measurement value was 0.92 and Bartlett’s sphericity was statistically significant ($$ {x}^{2}$$ = 4908.858, df = 1128, *p* <.001), thus the collected data were suitable for factor analysis. Communality was set at 0.30 to construct items that can express the meaning of factors well. The eigenvalue of ≥ 1.0, which means the explanatory power of the factors, and the scree plot were referred to for the number of factors. Items with a factor loading of ≥ 0.30 were considered meaningful [19], so items with a factor loading of < 0.30 were deleted. This study extracted 12 factors with an eigenvalue of ≥ 1.0, but 4 factors were suitable for the point where the eigenvalue of the scree plot rapidly decreases as a result of checking the “elbow” point (Fig. 1). Therefore, the number of factors was fixed at 4, and the first factor analysis was performed. Finally, the scale was confirmed with 4 factors and 27 items derived from the results of 4 EFA rounds, with 87.52% cumulative explanatory power of the scale (Table 2). The difference between the content of the item and the factor load was considered for items with overlapping loads on both factors. Hence, item 5 was deleted, and items 21 and 23 were placed in factor 3 with a greater factor load. Item 2 was maintained factor 2 by reviewing the contents. Factor 1 has 22.02% explanatory power with 4 items, factor 2 has 24.06% explanatory power with 8 items, factor 3 has 20.88% explanatory power with 7 items, and factor 4 has 20.55% explanatory power with 8 items.

**Table 2:** Rotated factor pattern matrix (28 items) (*N* = 273)

No	Items	Communality	Factors			
			1	2	3	4
8	Write important patient-related information appropriately in the standardized nursing record form or writing method.	0.47	**0.67**	-0.04	0.10	-0.02
6	Knows what information to provide at handoff.	0.39	**0.61**	0.08	0.03	-0.11
9	Use the electronic medical record system skillfully to collect patient information.	0.48	**0.56**	0.16	0.02	0.02
7	Knows hand-off methods, such as the order of information delivery and the data used.	0.41	**0.49**	0.03	0.18	0.01
5	Know the purpose, side effects, and precautions of drugs administered to patients. *	0.35	**0.33**	**0.31**	-0.02	0.04
4	Know the purpose, method, and precautions of clinical tests performed on patients.	0.48	0.04	**0.67**	-0.02	0.00
18	Deliver accurate information about the patient’s condition.	0.35	-0.02	**0.59**	0.03	0.00
13	Identify and deliver information about changes in the patient’s condition.	0.47	-0.00	**0.56**	-0.02	0.20
2	Know the needed nursing activities following the nursing protocol.	0.47	**0.42**	**0.44**	-0.11	-0.03
11	Identify and deliver information about the patient’s general characteristics.	0.37	0.16	**0.43**	0.02	0.06
14	Identify and deliver information on nursing work-related to patient treatment and clinical tests.	0.36	0.24	**0.41**	-0.02	0.03
10	Collect information about patient care from relevant departments.	0.36	0.24	**0.35**	-0.01	0.10
3	Know the clinical significance of test results performed on patients.	0.31	0.07	**0.34**	0.17	0.07
41	Structured by integrating data related to health issues, rather than listing information.	0.47	0.10	-0.23	**0.71**	0.07
21	Understand integratively patient’s health problems through synthesizing related data.	0.58	-0.25	**0.35**	**0.65**	0.01
40	Explain information related to health problems according to a causal relationship.	0.50	0.21	-0.18	**0.60**	0.14
22	Interpret the clinical significance of test results related to changes in the patient’s condition.	0.41	0.14	0.10	**0.50**	-0.04
23	Explain the patient’s health problem by identifying the contextual factors (cause, effect) related to the change in the patient’s condition.	0.38	-0.05	**0.33**	**0.46**	-0.13
20	Identify overall changes in the patient’s health problems.	0.44	0.14	0.29	**0.42**	-0.13
25	Prioritize nursing activities based on scientific evidence.	0.32	0.19	0.07	**0.33**	0.07
28	Discuss matters that are not understood concerning nursing tasks.	0.36	-0.09	0.01	0.00	**0.64**
36	Have conversation etiquette in tone and posture.	0.37	-0.04	0.07	-0.03	**0.60**
32	Positively accept any questions or feedback from the nurse (receiving nurse).	0.38	0.17	-0.17	0.05	**0.58**
27	Carry out handoff with a bright and positive attitude.	0.33	-0.03	0.08	-0.05	**0.56**
33	Empathize with the difficulties of the receiving nurses and encourage them to practice nursing in the future.	0.40	-0.09	0.07	0.14	**0.55**
31	The nurse who gives the handoff (sending nurse) provides an opportunity to ask questions to the nurse who accepts the handoff (receiving nurse).	0.38	0.13	0.03	-0.05	**0.55**
30	Discuss and seek advice if the nurse had experienced a difficult clinical situation.	0.31	-0.18	0.03	0.14	**0.54**
35	Politely excuse the receiver for any incomplete work or mistakes.	0.31	0.10	0.15	-0.11	**0.46**
Eigenvalue			1.40	1.90	1.15	9.09
Explained variance (%)			22.02	24.06	20.88	20.55
Total explained variance (%)			87.52			

*Bold values indicate items loading on factors

##### Confirmatory factor analysis

CFA was conducted to confirm the structural suitability of 27 questions and 4 factors derived through EFA. Maximum likelihood estimation was used for CFA, and a standard of ≥ 0.50 was applied for the standardization coefficient [21]. The contents were reviewed among the five items with a standardized coefficient of ≤ 0.50 for the first CFA, wherein two items (Items 27 and 33) were deleted. The second CFA result is shown in (Table 3). The fitness indices of the model were as follow: $$ {x}^{2}$$= 349.335 (*p* =.001), SRMR of 0.05, RMSEA of 0.04, TLI of 0.95, and CFI of 0.96.

**Table 3:** Results of confirmatory factor analysis (*N* = 223)

Factor	Items	Standardized estimates	SE	Critical ratio	CR
1	H6	0.68***			0.73
	H7	0.71***	0.13	8.71	
	H8	0.50***	0.14	6.45	
	H9	0.64***	0.12	7.97	
2	H2	0.62***			0.85
	H3	0.67***	0.15	8.22	
	H4	0.66***	0.13	8.11	
	H10	0.53***	0.12	6.78	
	H11	0.64***	0.11	7.92	
	H13	0.64***	0.12	7.89	
	H14	0.70***	0.13	8.45	
	H18	0.71***	0.14	8.53	
3	H20	0.68***			0.84
	H21	0.73***	0.12	9.48	
	H22	0.64***	0.12	8.49	
	H23	0.69***	0.13	9.01	
	H25	0.63***	0.12	8.33	
	H40	0.60***	0.12	7.96	
	H41	0.63***	0.13	8.32	
4	H28	0.52***			0.69
	H30	0.56***	0.21	5.66	
	H31	0.51***	0.18	5.31	
	H32	0.55***	0.18	5.57	
	H35	0.49***	0.17	5.18	
	H36	0.47***	0.18	5.05	
Fitness index	*X*^2^/*df*	SRMR	RMSEA	TLI	CFI
Reference value		≤ 0.08	≤ 0.08	≥ 0.90	≥ 0.90
Model (25 items)	349.335/269	0.049	0.036	0.959	0.964

#### Criterion-related validity

The criterion validity test revealed a significant correlation between the total score of the Nursing Handoff Competency Scale developed in this study and the scores of the NCRS and GICC-15 (*r* =.60, *p* <.01; *r* =.42, *p* <.01) The criterion validity of the scale developed in this study was verified (Table 4).

**Table 4:** Correlation between the nursing handoff competency scale and the nurses clinical reasoning scale (NCRS) and the global interpersonal communication competence (GICC-15) (*N* = 223)

	Nursing handoff competency scale				
	Total score	Knowledge on handoff methods	Identification of patient information	Judgment and transfer of nursing situation	Formation of supportive relationships
	*r *(*p*)	*r *(*p*)	*r *(*p*)	*r *(*p*)	*r *(*p*)
NCRS	0.60 (< 0.001)	0.41 (< 0.001)	0.53 (< 0.001)	0.52 (< 0.001)	0.44 (< 0.001)
GICC-15	0.42 (< 0.001)	0.22 (0.001)	0.33 (< 0.001)	0.28 (< 0.001)	0.52 (< 0.001)

#### Convergent and discriminant validity

The construct reliability of each factor greater than 0.7 was taken as an indicator to evaluate convergent validity [22]. In this study, the construct reliability of each factor was 0.73 (factor 1), 0.85 (factor 2), 0.84 (factor 3), and 0.69 (factor 4). Additionally, discriminant validity proves the degree of difference between factors. If the confidence interval of the correlation coefficient between factors does not include 1.0, discriminant validity is judged to have been secured [23]. In this study, discriminant validity was verified because the confidence interval of the correlation coefficient between factors did not include 1.0, so the discriminant validity was verified (Table 5).

**Table 5:** Discriminant validity between factors

Factor	*r*	Standard error	Confidence interval of the correlation coefficient between factors
Factor1 - Factor2	0.82	0.02	0.78–0.86
Factor1 - Factor3	0.74	0.02	0.70–0.78
Factor1 - Factor4	0.63	0.02	0.60–0.66
Factor2 - Factor3	0.76	0.02	0.71–0.80
Factor3 - Factor4	0.71	0.02	0.67–0.75
Factor3 - Factor4	0.54	0.02	0.50–0.57

#### Reliability

Cronbach’s α of the whole scale was 0.91 and the Cronbach’s α of the factors ranged from 0.688 to 0.848. Cronbach’s α of each factor was as follows: knowledge on handoff methods 0.72, identification of patient information 0.85, judgement and transfer of nursing situation 0.84, and formation of supportive relationships 0.69. The correlation coefficient for split-half reliability was calculated by dividing the items into odd and even numbers and then applying them to the Spearman-Brown formula, and was calculated as 0.85, thereby verifying the reliability of the scale.

### Final measurement scale

The nursing handoff competency scale was finally confirmed through the validity and reliability verification of the developed scale. The scale consists of 25 items with a total of 4 factors: “knowledge on handoff methods (4 items),” “identification of patient information (8 items),” “judgment and transfer of nursing situation (7 items),” and “formation of supportive relationships (6 items).” The scale has a 4-point Likert scale consisting of strongly disagree (1), disagree (2), agree (3), and strongly agree (4), and the measurement range is 25–100 points. The finally confirmed nursing handoff competency scale is shown in Appendix 1.

### Cut-off score

The extended Angoff method was used to set the criterion score. The standard setters consisted of new nurses and nurses with experience as preceptor. In the first round, each standard setter assigned the score (0–4 points) needed for a minimumally competent examinee to pass, resulting in a score of 64.83. In the second round, scores were assigned again after discussion between standard setters based on the scores derived in the first round. The final score of 71.25 was derived by multiplying the average of the final assigned scores by the number of questions. The total score of the scale developed in this study was derived as an integer, but the criterion score was calculated as 71.25. When determining the criterion score, the result of applying the criterion score should be considered [20]. Since the nursing handoff competency scale developed in this study was to select nurses who need nursing handoff education, selecting a high score can provide many subjects with educational opportunities. Therefore, the sufficient/insufficient criterion score of this scale was determined as 72 points.

In total, 17.5% of them have insufficient nursing handoff competency with a score of < 72.0 (Table 6). Among the participants with < 1 year of clinical career, 56.0% had insufficient nursing handoff competencies, which was higher than 44.0% of participants who had sufficient competencies, considering the difference according to the clinical career, whereas 12.6% among the participants with ≥ 1 year of clinical career had insufficient nursing handoff competencies. In particular, Six of the participants with 1–3 years of clinical career had a very low nursing handoff competency score of < 50 points. This indicates that nurses who need handoff education should include not only new nurses but also experienced nurses.

**Table 6:** Sufficient/insufficient judgment result of participants according to the cut-off score (*N* = 223)

Characteristics	Categories	Nursing handoff competency					
		Sufficiency n(%) or M ± SD		Insufficiency n(%) or M ± SD			x^2^ or t (*p*)
Total		184	(82.5)	39	(17.5)		
Age(years)		30.30 ± 4.72		27.77 ± 4.87		3.031(0.003)	
	< 30	92	(75.4)	30	(24.6)	9.862(0.007)	
	30–39	88	(91.7)	8	(8.3)		
	≥ 40	4	(80.0)	1	(20.0)		
Clinical career (years)		58.56 ± 42.02		22.51 ± 22.89		7.512(< 0.001)	
	< 1	11	(44.0)	14	(56.0)	44.553(< 0.001)	
	1–3	53	(73.6)	19	(26.4)		
	3–5	43	(97.7)	1	(2.3)		
	5–10	62	(92.5)	5	(7.5)		
	≥ 10	15	(100.0)	0	(0.0)		

## Discussion

This study developed a scale to measure nurses’ handoff competency and verified its reliability and validity. For handoff, a preparation process is needed to determine the patient’s current condition. In this process, the patient’s health-related data, nursing care performed, current treatment status, and information on treatment plans are selected and organized, and this convey through clinical judgment. Another characteristic of nursing handoff is to efficiently convey the information so that the receiving nurse understands the patient’s situation within the given time. Therefore, in this study, in the process of deriving the conceptual framework of the scale, the items were derived by integrating the results of the content analysis of interviews with clinical nurses as well as the literature review of 26 studies. Through this, we derived items to evaluate understanding ability, such as ‘Identify the overall changes in the patient’s health problems (item 13)’, ‘Explain the patient’s health problem by identifying the contextual factors related to the change in the patient condition (item 16)’. In addition, when deriving items, items about communication skills to convey information efficiently were derived. However, through exploratory factor analysis, only two items were retained. This was not classified as a separate factor, but was combined with items about judgment of the nursing situation and organized into the factor ‘judgment and communication of the nursing situation’. So, the final scale consisted of 4 factors and 25 items.

The first factor “Knowledge on handoff methods” includes four items pertinent to the type of information transferred during handoff, the method of handoff, documentation, and the use of an electronic medical record system. These items were derived based on the statements of new graduate nurses, who find handoff challenging, and experienced nurses with preceptor experience. Only 24.7% of facilities have written guidelines or a checklist for handoff within the unit although nurses receive handoff training after being hired [24, 25]. Consequently, most nurses receive only informal handoff training. Nurses who lack knowledge about handoff may omit important pieces of information or present irrelevant information [26, 27]. Unsystematic handoff causes prolonged handoff time and hinders follow-up nursing tasks [28]. Therefore, systematic handoff education and tools for identifying handoff education needs are required to enhance the efficiency of nurses in nursing tasks and help new graduate nurses adjust to clinical practice.

The second factor, “Identification of patient information” consists of eight items that assess whether important information about the patient’s health problems, treatment, and care was delivered during the handoff. The transfer of essential information about the patient’s health problems, treatment, and care between nurses at the change of shift is important to ensure nursing care continuity [29, 30]. Inadequate understanding of patient information engenders problems that threaten patient safety [31], and information omission and ambiguity hinder the follow-up care provided by the receiving nurse and thwart the provision of continuous care because the receiving nurse lacks knowledge about the current treatment status [32, 33]. Therefore, assessing whether a nurse has identified essential information and delivered them is important to measure handoff competency.

The third factor, “Judgment and transfer of nursing situation” comprises seven items about the clinical inference and judgment based on required patient information for a nursing handoff and the ability to transfer patient information. Critical thinking to understand the clinical situation is required during nursing handoff [34]. New graduate nurses experience difficulties to think critically regarding patient health problems even after a year in clinical practice [7]. Additionally, the item assessed to be the most difficult for the minimally competent person was “Comprehensively understanding the patient’s health problem by connecting relevant data” during the establishment of criteria for the scale in this study. The critical thinking process required for clinical judgment is essential to perform a handoff. However, existing handoff assessment tools only evaluate the handoff task itself and disregard the process of understanding the patient’s health problems before the handoff. Nurses’ handoff performance is assessed by determining their ability to analyse and understand the information in a given situation, and the developed scale is important because it contains items for this purpose. Moreover, the method of information delivery is an important part that affects the clinical decision-making of the receiving nurse [35]. This scale did not separate the competency to communicate effectively as a factor. However, the items “Explain the patient’s health problem by identifying the contextual factors (cause, effect) related to the change in patient condition (item 16)”, “Explain information related to health problems according to a causal relationship (item 18)”, and “Structured by integrating data related to health issues, rather than listing information (item 19)” are important items that measure the competency to deliver the context of the nursing process, rather than simply listing information.

The final factor, “Formation of supportive relationships” comprises six items pertinent to maintaining a collaborative and positive relationship between the transferring and receiving nurses. The quality of care is negatively affected by interpersonal problems among nurses, and nurses with better communication skills can communicate actively to promote patient safety [36]. A handoff can be categorized into technical communication, which involves the structuring and explanation of clinical information, and relational communication, which pertains to the interactions between the nurses [37]. The handoff quality is also influenced by relational factors, thus considering both aspects of communication are important. One of the items of our tool pertains to allowing the receiving nurse to ask questions and discuss difficult clinical situations together. The individual interviews with clinical nurses conducted in this study confirmed that the receiving nurse should check the patient’s condition and treatment status during handoff, and in the process, the receiving nurse could make up for the lacking parts of the sending nurse. Therefore a successful transfer of information requires a mutually respectful attitude between the two involved parties as well as evaluation and feedback to foster a supportive relationship.

As previously discussed, a key strength of the tool developed in this study is that it reflects the interpersonal relationship between nurses during a handoff as well as the nursing handoff. Existing instruments to evaluate the effects of handoff training do not reflect the purpose of a handoff, which is to help the receiving nurse understand the patient’s situation. Therefore, these instruments could not assess the process of summarizing and structuring patient data to transfer this information. The tool developed in this study contains items to assess the ability to understand and deliver data, reflects the nature of the nursing handoff based on field data obtained through interviews, and assesses the competencies required for a handoff comprehensively. However, Although this scale is suitable for inter-shift handoffs, further research is needed to determine whether it is also valid for interdepartmental handoffs.

In this study, we provided evidence on how well this scale reflects the concept it is intended to measure through verification of content validity, construct validity, and criterion validity. In scale validation research, the basis for validity cannot be sufficiently supported with just one approach, so various logical grounds must be presented [38]. This study is significant in that it presented a rational basis through various test of the validity of the scale. Moreover, another significant aspect of our scale is that we set cut-off scores to determine adequate/inadequate handoff competency. The cut-off score was set to 72 points. This score can be used to determine whether a nurse has adequate competency to perform a handoff. Among the second round study participants, 17.5% scored below 72, and the differences in the scores were significantly associated with the length of their clinical career. Also the results indicate that some experienced nurses also need additional handoff training, as identified by the cut-off score. This cut-off score is believed to expand the applicability of this tool. Healthcare facilities could determine nurses’ current handoff competencies and identify those in need of further training using this tool. The results of the tool will highlight the areas of weakness among nurses and hence can be useful for structuring relevant handoff training. Furthermore, handoff competency changes based on different career lengths and can be used as evidence data for handoff training for clinical nurses and as foundational data for developing a systematic handoff education system.

### Limitations

The nursing handoff competency measurement scale developed in this study is a self-assessment scale, thus the measurement result and the objective nursing handoff competency may be different depending on the participant’s perception. Therefore, the evaluation results of the receiving nurse, peers, nursing educators, and nursing managers should be considered together to objectively utilize the results of the nursing handoff competency measurement scale developed in this study. Additionally, more than 70% of the participants in this study were nurses from tertiary general hospitals and general hospitals and the number of care hospital nurses is low at 2.2%. Therefore, further study is needed to confirm whether it is appropriate to apply to care hospital nurses. Finally, the convergent validity verification results for this tool did not meet the validity criteria. Therefore, further research is needed to address this issue.

## Conclusions

The nursing handoff competency scale developed in this study is a consistent and valid evaluation tool. It consisted of 4 factors and 25 items and was a self-assessment tool on a 1–4 point Likert scale. The scores range from 25 to 100, and higher scores indicate higher nursing handoff competency. A score of ≥ 72 can be interpreted as sufficient nursing handoff competency. Therefore, we recommend this scale to evaluate the competency level to perform nurse handover, determine the need for educational support, and check the effectiveness of education.

### Electronic supplementary material

Below is the link to the electronic supplementary material.

Supplementary Material 1

## Data Availability

The datasets used or analyzed during the current study are available from the corresponding author on reasonable request.
