# Supplementary material for: Development of nursing handoff competency scale: a methodological study
Source: BMC Nurs. 2024 Apr 24;23:272. doi: 10.1186/s12912-024-01925-w (PMC11044331; doi:10.1186/s12912-024-01925-w)
Supplement: Supplementary file 1 — Supplementary Material 1 [file 12912_2024_1925_MOESM1_ESM.docx]

# Appendix 1. Nursing Handoff Competency Scale

| item | | Strongly disagree | disagree | agree | Strongly agree |
| --- | --- | --- | --- | --- | --- |
| 1 | Knows what information to provide at handoff. | ① | ② | ③ | ④ |
| 2 | Knows handoff methods, such as the technique of information delivery and the data used. | ① | ② | ③ | ④ |
| 3 | Write important patient-related information appropriately in the standardized nursing record form or writing method. | ① | ② | ③ | ④ |
| 4 | Use the electronic medical record system skillfully to collect patient information. | ① | ② | ③ | ④ |
| 5 | Know the needed nursing activities following the nursing protocol. | ① | ② | ③ | ④ |
| 6 | Grasp the clinical significance of the patient’s clinical test results. | ① | ② | ③ | ④ |
| 7 | Know the purpose, method, and precautions of clinical tests performed on patients. | ① | ② | ③ | ④ |
| 8 | Collect information about patient care from relevant departments. | ① | ② | ③ | ④ |
| 9 | Identify and deliver information about the patient’s general characteristics. | ① | ② | ③ | ④ |
| 10 | Identify and deliver information about changes in the patient’s condition. | ① | ② | ③ | ④ |
| 11 | Identify and deliver information on nursing work-related to patient treatment and clinical tests. | ① | ② | ③ | ④ |
| 12 | Provide accurate information about the patient’s condition. | ① | ② | ③ | ④ |
| 13 | Identify the overall changes in the patient’s health problems. | ① | ② | ③ | ④ |
| 14 | Understand integratively patient’s health problems through synthesizing related data. | ① | ② | ③ | ④ |
| 15 | Interpret the significance of clinical test results related to changes in the patient’s condition. | ① | ② | ③ | ④ |
| 16 | Explain the patient’s health problem by identifying the contextual factors (cause, effect) related to the change in the patient condition. | ① | ② | ③ | ④ |
| 17 | Prioritize nursing activities based on scientific evidence. | ① | ② | ③ | ④ |
| 18 | Explain information related to health problems according to a causal relationship. | ① | ② | ③ | ④ |
| 19 | Structured by integrating data related to health issues, rather than listing information. | ① | ② | ③ | ④ |
| 20 | Discuss matters that are not understood concerning nursing tasks. | ① | ② | ③ | ④ |
| 21 | Discuss and seek advice if the nurse has experienced a difficult clinical situation. | ① | ② | ③ | ④ |
| 22 | The nurse who gives the handoff (sending nurse) provides an opportunity to ask questions to the nurse who accepts the handoff (receiving nurse). | ① | ② | ③ | ④ |
| 23 | Positively accept any questions or feedback from the nurse (receiving nurse). | ① | ② | ③ | ④ |
| 24 | Politely excuse the receiver for any incomplete work or mistakes. | ① | ② | ③ | ④ |
| 25 | Have conversation etiquette in tone and posture. | ① | ② | ③ | ④ |
